# Phage Therapy Regulation: From Night to Dawn

**DOI:** 10.3390/v11040352

**Published:** 2019-04-17

**Authors:** Alan Fauconnier

**Affiliations:** Culture In Vivo ASBL, rue du Progrès, 4, boîte 7, 1400 Nivelles, Belgium; alan.fauconnier@invivo.be

**Keywords:** phage therapy, PTMP, ATMP, regulatory framework, pharmaceutical paradigm shift, clinical trial, magistral formula, personalized medicine

## Abstract

After decades of disregard in the Western world, phage therapy is witnessing a return of interest. However, the pharmaceutical legislation that has since been implemented is basically designed for regulating industrially-made pharmaceuticals, devoid of any patient customization and intended for large-scale distribution. Accordingly, the resulting regulatory framework is hardly reconcilable with the concept of sustainable phage therapy, involving tailor-made medicinal products in the global perspective of both evolutionary and personalized medicine. The repeated appeal for a dedicated regulatory framework has not been heard by the European legislature, which, in this matter, features a strong resistance to change despite the precedent of the unhindered implementation of advanced therapy medicinal product (ATMPs) regulation. It is acknowledged that in many aspects, phage therapy medicinal products are quite unconventional pharmaceuticals and likely this lack of conformity to the canonical model hampered the development of a suitable regulatory pathway. However, the regulatory approaches of countries where phage therapy traditions and practice have never been abandoned are now being revisited by some Western countries, opening new avenues for phage therapy regulation. As a next step, supranational and international organizations are urged to take over the initiatives originally launched by national regulatory authorities.

The idea of using bacteriophages to cure patients originally emerged in d’Hérelle’s mind one hundred years ago [1]. However, in the middle of the last century, the introduction of antibiotics led to the banishment of phage therapy from mainstream medical practice, whereas it remained in use in Eastern Europe, for instance in several institutes in Russia, in the Eliava Institute of Bacteriophage, Microbiology and Virology in Tbilisi (Georgia) and in the Hirszfeld Institute in Wroclaw (Poland). After decades of neglect in the Western world, phage therapy has witnessed a remarkable return to interest, as evidenced by the profile of PubMed search results, which features an increase in the late nineties to the start of the millennium [2]. This renewed interest is essentially due to the growing incidence of antibiotic resistance. However, this re-emerging therapy now faces the regulation that has been implemented since the days of d’Hérelle, entailing serious difficulties. Modern pharmaceutical legislation has been pointed out as a hindrance to phage therapy implementation [3] and has been consistently blamed for obstructing its deployment [4,5,6,7]. The regulatory issue impacts not only the market placement but also the conduct of clinical trials.

For these reasons, the phage community has called for a switch in the mindset of regulatory agencies [7] or even in the pharmaceutical paradigm [6,8]. Clearly, phage therapy turns the conventional rules and established codes upside down. For instance, because bacteriophages are self-replicating, phage therapy has been reported as an active treatment, referring to a concept developed in agricultural biocontrol [9]. This means that the drug may amplify in the body, depending on the bacterial density, which, in turn, is evolving in response to the phage density. This makes phages fundamentally different from passive pharmaceuticals such as antibiotics, whose concentrations decline by a combination of metabolism or excretion processes, according to more canonical pharmacokinetic behaviors. The pharmacology of phage therapy is thus quite unusual [9,10] and, according to models, might give rise to unexpected therapeutic outcomes such as a reduction or failure of efficacy when inoculation is given too early or because of the adjuvant use of antibiotics [11]. Phage therapy also differentiates in the evolutionary considerations it elicits. As observed with antibiotics, phage therapy will likely entail the selection of phage-resistant bacteria. However, given the narrow host range of phages as compared to the broader therapeutic spectrum of antibiotics, the selection pressure for resistance is exerted on only a limited number of bacterial types [12]. Moreover, it has been reported that phage resistance may reduce the virulence of bacteria [13,14]. Finally, phages are themselves evolving, giving rise to co-evolutionary dynamic patterns [15], which will never happen with antibiotics and poses a unique challenge for regulators [16]. The dynamics of the phage–bacterial interaction are becoming increasingly complex owing to the interference of a third intervener, which is the patient’s body. Thus, in contrast to the classical mechanistic approach of medicine, phage therapy will only reveal its full dimensions in a Darwinian medicine perspective, which takes evolutionist and ecological prospects into account [2,17,18]. Another specific feature of phage therapy relates to its economic viability. The current business model of pharmaceuticals, requiring large and costly randomized, double-blind clinical trials is hardly applicable to phage therapy. This clinical development, normally attainable for medicinal products used to treat chronic medical conditions, becomes tricky for “ordinary” antibacterial compounds intended to be used for short durations [19], and even more arduous for medicinal products made of natural phages, which can only benefit from limited intellectual property protection and whose poor return on investment would likely not balance the resource expenditure [2,7,20]. In this regard, a Supreme Court jurisprudence analyzing patentable subject matter questions the eligibility of phage therapeutics for strong patent protection [21,22]. In spite of this, patents covering the use of phage therapy have been granted [23] and some clinical trials have been conducted or are still ongoing in Europe [24,25,26] and the United States [27,28,29], but so far they have not contributed to any licensing. Given that no phage product is currently marketed, authorities have little incentive to develop regulatory schemes and guidelines specific to bacteriophages [30] and, through a negative feedback loop, the absence thereof constitutes a hindrance to phage therapy development. Further clinical evidence would thus help to foster regulatory advance. In the European Union (EU), investigational medicinal products (IMP) defined as “a pharmaceutical form of an active substance or placebo being tested or used as a reference in a clinical trial” must be manufactured and checked in compliance with the principles and guidelines of good manufacturing practice (GMP) [31]. However, GMP compliance represents a real challenge and requires extensive financial resources [16,25,32], which may constitute an insurmountable obstacle for phage therapy sponsors when they are hospitals or non-for-profit phage therapy centers.

Besides these atypical features, there is one additional singularity making phage therapy medicinal products (PTMPs) unconventional, namely their qualitative and quantitative composition, which may be subject to variations. Indeed, PTMPs are either ready-prepared medicines intended for large scale distribution or patient-specific, tailor-made preparations issued from local small-scale productions. While the former, which have a fixed composition, match the current regulatory framework, the latter, with their moving target formulation, do not. In Europe, the pharmaceutical legislation was basically launched in the early sixties, following the thalidomide tragedy. It was designed to control industrially-made pharmaceuticals. The amended European Directive 2001/83/EC related to medicinal products leaves no doubt in this regard since it applies to “medicinal products for human use intended to be placed on the market in Member States and either prepared industrially or manufactured by a method involving an industrial process” [33], as opposed to the “medicinal products prepared in a pharmacy in accordance with a medical prescription for an individual patient (commonly known as the magistral formula)”, which are beyond the scope of the directive. Still, in the early 2000s, autologous cell-based therapeutics first began to create problems in this regard. As they are patient-specific and given that some of them are prepared locally in the hospital pharmacy, they relate to the magistral formula. However, their manufacture may involve an industrial process, especially when part of their manufacturing process takes place in a biotech company. As such, they should fall within the scope of Directive 2001/83/EC. Moreover, assessing the quality and the benefit/risk balance of these innovative therapeutics is sensitive, thus amply justifying their tight regulatory control and, accordingly, their licensing as prescribed in the directive. To overcome this contradiction, the European legislature coined the concept of advanced therapy medicinal products (ATMPs) and designed a specific regulatory framework. Autologous somatic cell therapy medicinal products and tissue engineered products are both ATMPs. Strictly speaking, these medicinal products change from one patient to another since they stem from a patient’s own cells, although they share a common manufacturing process. Therefore, the basis on which the marketing authorization is issued switched, and instead of focusing on the product itself, it became process-driven. Interestingly, the extent of quality, non-clinical and clinical data to be included in the marketing authorization application may be determined using a risk-based approach. Furthermore, ATMPs “which are prepared on a nonroutine basis according to specific quality standards, and used within the same Member State in a hospital under the exclusive professional responsibility of a medical practitioner, in order to comply with an individual medical prescription for a custom-made product for an individual patient” [33] may be discharged of the marketing authorization obligation under the umbrella of the so-called “hospital exemption” procedure. The similarity to phage therapy is undeniable. Custom-made PTMPs have more to do with cottage factories than with big pharma, as they are patient-specific, and, at the same time, they may share a common industrial process. This analogy did not escape the attention of regulators [16] and researchers engaged in the field, who advocate for a specific regulatory framework for phage therapy [34,35,36] that could, for instance, take advantage of the hospital exemption [37].

Autologous ATMPs are not the only pharmaceuticals that face a conflict between tailor-made production and industrial manufacturing. For instance, custom-made, anti-sense, oligonucleotide medicinal products also fall between the two. Linked to this, a call for new pharmaceutical legislation has been issued [38]. Similarly, as observed in cancer management, the concept of large disease groups being administered ”one-size-fits-all” blockbuster drugs is gradually being replaced by the stratification of patients into small sub-groups, each treated with a different medication [39]. These medicinal products, as well as the PTMPs, share with autologous ATMPs the fact that they relate to personalized medicine. However, they are not considered ATMPs, and therefore they cannot benefit from the exceptions foreseen for ATMPs [16].

Encompassing phage therapy within personalized medicines is a direct consequence of the narrow therapeutic spectrum of bacteriophages. However, while high specificity has its advantages, it also carries drawbacks [16]. Prior to patient treatment, the phage susceptibility of the infecting bacteria must be determined by performing a phagogram. The expected time frame for performing such an analysis is expected to be similar to the turn-around-time for antibiogram results. However, the selected phage(s) must then be amplified, a process that may take an additional 18 h [40,41]. This can make a difference in the management of a bacterial infection.

In view of the underlying trend toward personalized medicine, the difference in the regulatory treatment of the custom-made medicines is difficult to understand. Indeed, whereas the European legislature implemented rather quickly the specific regulation for ATMPs, it has since showed a defensive position resistant to regulatory change. The design of the adaptive pathway is an instructive example in this respect [42]. To meet the need of critically ill patients, the European Medicines Agency (EMA) implemented this pathway, which relies on the procedures of scientific advice, compassionate use, conditional approval mechanism, and pharmacovigilance tools. Interestingly, it is clearly mentioned that this approach makes use of regulatory processes already in place within the existing EU legal framework. No new regulatory pathway has been implemented. The EMA therefore had to develop a creative regulatory avenue, while respecting the status quo attitude of the European lawmaker. However, in terms of regulatory affairs, the most intense creativity has its legal limitations. Thus, a workshop organized by the EMA in 2015, aimed at facilitating the development of bacteriophage therapy by reviewing regulatory aspects, eventually failed to deliver tangible openness towards an alternate regulatory scheme [16], primarily because of the EU decision-makers’ conservatism. The European Commission made clear that the existing regulatory framework is adequate for bacteriophage therapy and that PTMPs can be regulated like any other medicinal product [43], whereas the stakeholders repeatedly expressed their disagreement with this stance [7]. It has thus proved necessary to turn to the Member States to find the beginnings of a solution.

Recently, the Belgian authorities have opened a gateway to phage therapy regulation by taking advantage of the national regulation of magistral preparation (compounding pharmacy in the US) [44]. The procedure relies on two cornerstones, namely (i) the issuing of a monograph serving as a written standard for assessing the quality of the phage active substance to be used as raw material for the preparation of the PTMP, and (ii) the availability of a Belgian approved laboratory that is able to test the phage stock and, where applicable, may issue a certificate of analysis stating that the tested phage complies with the monograph, in line with the current state of technical and scientific knowledge. The pharmacist can then use this certified material for preparing a customized medicinal product based on the prescription of a physician. This regulatory scheme is probably not optimal, since it places all the responsibility on the prescriber and the pharmacist, exempting the manufacturers and the regulatory authorities from the liability that they normally have for authorized medicinal products [36]. Therefore, it should be regarded as transitional [45]. However, even though it has some shortcomings, this process has at least the virtue of existing and of breaking down the regulatory barrier. As such, it was welcomed as a breakthrough that nurtures hope for the implementation of phage therapy in accepted therapeutic practices [46]. Changes are also taking place in France, where a specialized scientific temporary committee on phage therapy issued recommendations for using PTMPs under the umbrella of the so-called nominative Temporary Authorization for Use (ATUn, standing for Authorisation Temporaire d’Utilisation nominative) subject to certain conditions [47]. The ATUn of a medicinal product is issued for a single named patient who cannot participate in a clinical trial, at the request and under the responsibility of the prescribing physician. The ATUn is an exceptional authorization procedure, issued by way of derogation, which allows, in the absence of any appropriate alternative treatment, a medicinal product with no marketing authorization to be made available provided that its efficacy/safety balance is presumed to be favorable for these patients based on the available data. Medicinal products with ATUns can only be dispensed by hospital pharmacies.

Regulatory change can also be identified across the Atlantic. In the United States, some patients were treated with phages following the emergency investigational new drug (eIND) pathway of the Food and Drug Administration (FDA) [48,49]. Indeed, patients may have access to non-approved drugs or biological products under the expanded access program. Among the different categories of expanded access, the individual patient expanded access IND for emergency use appeared suitable for personalized phage products, which are regulated as biologics in the jurisdiction of the Office of Vaccines Research Review, in the FDA Center for Biologics Evaluation and Research (FDA/CBER/OVRR).

Interestingly, the Western world now implements regulatory principles that are reminiscent of the ones that apply in the countries where phage therapy traditions and practice have never been abandoned. In Georgia, regarded as a stronghold for bacteriophage therapy [50], phage products are considered to be pharmaceuticals. Bacteriophage ready-to-use medicines require a marketing authorization according to regular legislation. As for customized phage preparations, they may be prepared as a magistral preparation in an authorized pharmacy that has been granted a special license issued by the Georgian Ministry of Healthcare on the preparation of extempore medications. In Russia, which also has a longstanding practice of phage therapy, there is a precedent for the Belgian monograph [51], since the Russian pharmacopeia includes a monograph on bacteriophages for prophylactic and therapeutic use [52].

Phage therapy is not only moving forward in receiving regulatory approval. Progress may also be expected in clinical trial applications. With this in mind, it is worth mentioning that there is a new provision in the EU regulatory framework that may have gone unnoticed by the phage therapy sponsors, although it could facilitate PTMP clinical development. Whereas the former EU provisions relating to the conduct of clinical trials prescribe that the principles of GMP should be applied to IMP [15], some flexibility is foreseen in Regulation 536/2014, repealing Directive 2001/20/EC [53]. Indeed, according to Art 61(5) and 63 of this regulation, the preparation of IMPs “where this process is carried out in hospitals, health centres or clinics legally authorised in the Member State concerned to carry out such process and if the IMPs are intended to be used exclusively in hospitals, health centres or clinics taking part in the same clinical trial in the same Member State” may be exempted from GMP requirements. This provision markedly reshapes the EU landscape of clinical trial applications and may help meet the repeated demand for scientific evidence from human trials conducted to modern standards [16].

The regulation of PTMPs is evolving slowly but is moving in the right direction. The appeal for a paradigm change is beginning to be heard at least at the national level where recent initiatives are overcoming regulatory obstacles to a certain extent. However, despite this progress, there is still a way to go before a fully practicable regulation is implemented. The next step might come from international organizations. In the European Union, an initiative needs to be taken at the Community level to provide a genuine and harmonized regulation for PTMPs. In more general terms, considering the profound changes occurring in therapeutic practices, and especially the increasing personalization of medicine, it is the author’s opinion that the EU lawmakers can no longer maintain their position resisting change without facing the risk of hampering innovation and, more critically, ignoring patients’ needs. At a higher regional level, the Council of Europe’s Directorate for the Quality of Medicines and Health Care could also be involved through the European Pharmacopoeia (Ph.Eur.). Indeed, elaborating a Ph.Eur. text on phage therapy would foster harmonization and strengthen the scientific base of what would then become an official public standard. Lastly, at a global level, the involvement of the World Health Organization appears essential for the development of phage therapy in general [54], and especially for its implementation in the low- and middle-income countries where it is urgently needed [55].

To conclude, it is worth emphasizing that the issue of PTMP regulation extends well beyond the area of phage therapy, since the debate is fundamentally related to the customization of medicinal products tailored to an individual patient. From this perspective, we like to think that while bacteriophages were a prominent model for uncovering the nature of genes, they remain so in the separate but promising context of personalized medicine.
